# Book review: Integrity in education for future happiness

**DOI:** 10.1007/s40979-021-00076-8

**Published:** 2021-04-02

**Authors:** Irene Glendinning

**Affiliations:** Coventry University, Priory Street, Coventry, CV1 5FB UK

**Keywords:** Conference proceedings, Academic integrity, Integrity in ethics and research

## Abstract

The joy of doing any review is having a legitimate excuse to spend time learning about what other researchers are working on, then to reflect on what is new. This book review with a rich tapestry of current research and thinking about integrity in education and research, is no exception. This publication is the product of a virtual conference that took place in April 2020, which, had the Covid-19 pandemic not arrived, would have been held face-to-face in Dubai.

Each of the 15 chapters brings to light new ideas for encouraging academic integrity and ethical conduct, or for deterring and managing academic dishonesty. Some chapters place familiar problems and solutions into new contexts and illustrate classroom experiences in different parts of the world. The key topic of education and training of teachers, researchers and students features in several chapters, including a welcome focus on secondary education. There is no shortage of empirical research in the book, including analysis of data on institutional policies an, admissions. Three chapters concern innovations in use of technology and how they are being applied and developed, with useful take-aways for evidencing contract cheating.

Inclusion of the student voice is important in research into academic integrity. We hear from students through formal research, informal feedback and a whole chapter about one student’s journey.

The book has been fittingly dedicated to the memory of Tracey Bretag (19/6/1962–7/10/2020), who managed to continue her support for the series of PAEB conferences by recording her keynote, despite being already very frail. Whether you decide to read the whole book or just select a few chapters, the ideas you will find there largely stem from Tracey’s legacy. We are reminded that there is so much more to do to improve integrity in education and research, locally and globally.

This is no ordinary book. I will try to convey why I believe the book is special during this review, but before I set out my thoughts, it is important that I declare my conflicts of interest. As former vice-president for the European Network for Academic Integrity (ENAI) and a long-standing researcher, supporter and follower of research in this field, most of the authors who have contributed to this book are my colleagues and friends. I am aware of their commitment to academic integrity and all the excellent work they have done already. I have worked and co-authored with many of these researchers in the past. I come to this review as an active participant in the conference from which this book was generated. As a native of Yorkshire, I am renown for my straight-talking, but I do not wish to offend my friends and colleagues, past, present and future. Taking all these factors into account, it is very difficult for me to be frank and objective, but I will do my best to provide a full and fair critique of the book.

Academic integrity is becoming increasingly crucial for maintaining educational standards and quality. It is an ephemeral concept, as Sonja Bjelobaba discusses in the opening chapter focusing on teacher training (PAEB Dubai 2020: 9–18), academic integrity is too often defined negatively, in terms of what it is not. The material for this book is based on a conference that had been planned for 2 years before the 2020 global pandemic struck. The 6th Plagiarism Across Europe and Beyond conference, organised jointly by ENAI and University of Wollongong in Dubai, should have been held in Dubai in April 2020, but instead, with very little notice, the organisers took the brave decision to move it fully on-line, without rescheduling dates or content. As a keen participant in all six conferences to date, I can report that this was the correct decision. The conference was a triumph, a model for how to conduct a virtual event, technically, socially and academically. For more details about the conference, it is worth visiting the conference web site (PAEB 2020).

Each of the 15 chapters in this book illuminates how integrity applies to different aspects of education, what is being achieved in different settings, and the book as a whole underlines what more needs to be done. As with any conference the parallel sessions make it impossible to attend every interesting presentation on offer. This book provides the opportunity to find out about missed sessions, but also to recall what was presented and find more detail on the research behind presentations. The educational scope of topics includes a focus on secondary education, teacher training, discrepancies in admissions, English language teaching, ideas for teaching ethics to both biomedical and business students.

Given that the prevalence and nature of academic misconduct varies in different institutions and countries, responses to cheating must be designed according to the local needs. What works in one context does not always translate to all situations, but it is healthy to share experiences and to hear from others what worked and what was less successful. Accordingly, contributions from UAE, Hungary, Bangladesh, USA, UK, Japan, Slovakia, Sweden, Canada and Montenegro provide excellent geographical diversity, drawn from the global perspectives of all contributions during the conference.

Sadly, the Future Happiness promised in the title of this book does not feature in every chapter, but where it does emerge it is a welcome change from negative sentiments often expressed in writings on this subject. A “triage” system in a UAE university provides counselling for students who have had allegations of academic misconduct upheld, giving students an opportunity to express their emotions, address and correct their failings, then move on with their lives (PAEB Dubai 2020: 90–102). Another example is a comprehensive study on institutional policies on plagiarism in Hungary (PAEB Dubai 2020: 112–126), building on part of an earlier research study that I led on this topic (IPPHEAE 2013). Although Hungary still has a long way to go, I was delighted to see how much progress has been made in recent years and how seriously most higher education providers in Hungary are now taking this challenge.

Several chapters concern different aspects of the use of technology and software tools. The work by Crockett & Best on innovative application of “stylometrics” has potential for greatly improving the quality of evidence when contract cheating is suspected (PAEB Dubai 2020: 35–49). A chapter by Johnson & Davies explores how digital forensics can be used to help to detect and evidence contract cheating (PAEB Dubai 2020: 78–89). This study includes very useful technical details of how to gain access to Open Office XML format (OOXML) used in Microsoft Office documents, with examples of what can be learned from examining this source of evidence. These two chapters, from Crockett & Best and Johnson & Davies, report on very promising early research, which, hopefully they will continue to develop.

Daniel Dusza’s chapter presents a series of four experiments to monitor, analyse and verify the creative writing process, “to improve the integrity of authored material” (PAEB Dubai 2020: 51). The ultimate component of this research involved using technology for “covertly” monitoring Japanese students, writing in a second language, completing a 1000-word essay and related activities. Putting aside questions about how much the students were aware they were being monitored, also whether there was ethical approval and informed consent for participants involved in this study, there are some interesting observations about what was discovered about student conduct in the course of this study. The four experiments provide useful information about how teachers can improve the way students are prepared for academic writing tasks. The author concludes with suggestions for how background monitoring technology could be used in future to intervene if students appear to be at risk of violating academic integrity while they are writing. Although this chapter is about “big data”, to me the last element also is worryingly reminiscent of “big brother”. In my view, using technology for proctoring students in the way described, risks breaching personal privacy, therefore needs prior consent and buy-in from the students, whether use is experimental or operational.

When we conducted research across the 27 European Union (EU) countries for the IPPHEAE project in 2010–13, on the top of the wish list of many EU countries were the need for affordable “anti-plagiarism” software and a national corpus of academic publications, dissertations and theses, predominantly focusing on one language. At that time Slovakia stood out from the crowd as it already had an effective national system in place (IPPHEAE 2013). Julius Kravjar’s chapter describes how SK ANTIPLAG was developed by The Slovak Centre of Scientific and Technical Information (SCTI) and introduced nationally as a central system in April 2010 (PAEB Dubai 2020: 103–111). It was good to hear that a similar system was introduced in Poland in 2019, in addition to those in place in Czechia and Slovenia. Kravjar describes how the 10 years of data now accumulated in the Slovakian repository provides a rich resource for interesting analysis. The author explains that the key to the data capture is the quality of the metadata stored with each document and provides some examples of analyses and insights that are possible.

It is my view that there are missed opportunities if national systems of this type are just used in isolation, for storing and checking theses and dissertations, rather than checking all student assignments and sharing the data internationally. Clearly sharing data would depend on compatibility between the national systems. The advantage of checking all assignments is the need to nurture students’ study and academic writing skills from the earliest opportunity, developing their expertise throughout their studies, rather than leaving the checking of their skills until the “high stake” final thesis. Although text matching software is not a silver bullet for eliminating plagiarism, it’s more positive use is for teaching academic writing skills, if harnessed as an effective part of the learning process.

There is an increasing awareness that education for academic integrity needs to begin before students enter higher education. Too often students arrive at university lacking understanding of concepts such as intellectual property rights, how to make use of information sources created by other people. The chapter from Bangladeshi authors Shaha, Hossain & Asaduzzman, reminds us how fortunate teachers and students are in many countries, just to have a reliable supply of electricity as well as up-to-date technological equipment and software, that we often take for-granted (PAEB Dubai 2020: 166–177). It transpires that secondary teachers of English in Bangladesh often resort to using free “plagiarism checking” services to check students’ work. These free services are generally not to be recommended, because they are often ineffective and can be associated with essay-mills. The authors conclude with a checklist of urgent requirements for Bangladesh, including the need for teacher training, particularly on managing and using digital content.

Staying on the subject of teacher training, in the first chapter of the book, Sonja Bjelobaba shares her experience of running workshops for training higher education teachers, in Sweden and elsewhere, on pedagogical approaches to academic integrity (PAEB Dubai 2020: 9–18). The chapter includes some very helpful ideas for learning outcomes, content and structure relating to academic integrity. I was delighted to note that the author advocates use of Bloom’s taxonomy, an old favourite of mine that I still use in workshops with both teachers and students, for differentiating between different levels of education, in designing learning outcomes and assessment criteria.

Rakhman & Khan from UAE conducted a study focusing on a neglected area of academic integrity research, relating to student admissions to higher education (PAEB Dubai 2020: 90–102), but I was disappointed in how limited the data collection methods were. The research relied only on published criteria on academic qualifications from four UAE universities for undergraduate admission, leading to discussion of inconsistencies between institutional practices. Although this is a useful start, there is so much more that could be achieved in this area of research, affecting public and private education in all parts of the world. For example, how often are students admitted without the required qualifications, if this happens, under what circumstances, perhaps (positively) accreditation for prior learning and experience (APEL) or (negatively) favouritism, bribery and corruption? There was no mention of about other forms of credentials, such as evidence of language skills, which can be fake or obtained fraudulently. Is there any evidence linking poor performance and academic misconduct with standards and criteria used for higher education admission? Are students who are admitted with weak qualifications more prone to cheating than those with strong credentials? What measures are taken to detect and deal with fraudulent evidence and fake credentials presented by applicants? This topic is crying out for further in-depth research.

There have been many previous discussions and research studies about what makes students plagiarise and cheat, but I found new ideas in the chapter on this topic from a team of researchers in Montenegro, it is well worth reading (PAEB Dubai 2020: 127–154). The authors took the Extended Theory of Planned Behaviour (ETPB) and extended it still further, not only to show evidence of influences to behaviour, but also under what circumstances behaviours were moderated by, for example, introducing similarity checking software. It would be interesting to repeat this research in other places.

I was very interested to read the study by Sivasubramaniam & Khan (PAEB Dubai 2020: 186–194) comparing institutional procedures for handling academic misconduct by students at five UK universities. This research reveals the disparity and variety of practices, even in this small study within one country. The authors are rightly looking for evidence of fairness, consistency, proportionality of both process and outcomes and their conclusions are generally in keeping with the evidence presented. One criticism I have is that the specific circumstances, such as size and geography of the universities, which can vary greatly, have not been factored in before judgements are stated or implied by the authors. It makes sense to have a centralised panel for consistency, with a single campus institution of 5000 students, but this is less feasible if, for example, there are multiple campuses, in various locations and a student population of, say, over 25,000 students. The authors point to worrying evidence in this study that in at least one of the universities the Turnitin similarity percentage serves as a discriminator for escalating cases with higher so-called “plagiarism percentages” to a more senior level panel. If, as proposed by the authors, this study is expanded it would be valuable to have direct involvement from the universities concerned to reveal what happens in practice within each institution, rather than relying on documentary sources. This direct contact would provide the opportunity for dialogue between the researchers and the institutions to encourage improvements where warranted.

Providing real examples of ethical dilemmas can help bio-medical students to become inspired and engaged when learning about ethical conduct and decision-making. Sivasubramaniam & Khan based their other co-authored chapter on a workshop they ran for PhD students after the ENAI conference in April 2020 (PAEB Dubai 2020: 178–194). Moving the workshop online meant that discussions on the six ethical examples presented could not take place face-to-face, but despite this handicap, there were so many questions and interactions from the student participants that the workshop lasted a full hour longer than scheduled. The authors have included very rich feedback in this chapter from the participants, which can provide useful ideas for others involved in delivering this subject.

Caroline Burns provides an alternative approach to teaching ethics by using axiology, based on self-reflection by students to explore their values and motivations in the context of a business ethics module (PAEB Dubai 2020: 19–34), which should impact on their future professional conduct. The penultimate class of the module focuses on “Determining the relationship between each student’s values and their attitudes toward academic integrity and academic misconduct.” The final class links ethical conduct in education with what the students will need for the workplace. I found the “Anecdotal Observations” about the students particularly enlightening (PAEB Dubai 2020: 25–26), including views about the inevitability of cheating and the ways that both workplace and educational cheating could be justified by the students.

The innovative approaches described here for teaching ethics, from Sivasubramaniam & Khan and Burns, should be of broader interest, because both could be readily adapted for delivery to students from other disciplines.

The last chapter in the book, a first-hand account of one student’s experience, brings us back to the book’s title and future happiness (PAEB Dubai 2020: 195–205). It tells the story of how the student gained an understanding of academic integrity, gradually building on her earlier experiences. The authors use a simple table to encapsulate the student journey, summarised in the rows as MAP: Moral System, Attitude and Pressure, with the columns indicating how this played out over time in school, university and the workplace (PAEB Dubai 2020: 202). For me this chapter contains three important messages: the need for educational policy-makers to listen to the views of students; to have effective delivery of education about academic integrity throughout the student journey and beyond; and that is never too early to talk to young people, or too late to talk to young professionals, about the advantages of behaving with integrity.

My main criticism of the book is inattention to detail in places, in terms of proof-reading. I found some simple errors that perhaps should have been noticed and in a few chapters the lack of fluency in the English was quite distracting, but the editors have agreed to add corrections to the on-line version. In the meantime, hopefully, occasional grammatical mistakes and typos will not detract too much from the experience of reading the book. The positive aspects of the book far outweigh the negatives.

At the start of the book is a fitting dedication from the publishers, editors and authors to Professor Tracey Bretag, who, as many readers will know, sadly died in October 2020. Tracey has been a friend and mentor to many of the people involved in producing this book and an inspiration to those of us throughout the world who are interested in integrity in education. Her keynotes for the five previous conferences were thoughtful and innovative. She did not disappoint in her last contribution. She was too ill even to join us on-line in April 2020, but took the time to record her final message to the conference in advance, which can still be accessed and is well worth watching (Bretag 2020). She is already missed very much by the global community of researchers in this field, but her legacy of research and powerful inspirational messages and principles will continue to drive us all forward.

Although a visit to Dubai in April 2020 would have been wonderful, this book is a fitting record of the on-line event. In case you are not already convinced, I believe the book is exceptional because, apart from the very fine contributions reviewed above, it demonstrates the courage of the conference organisers, regardless of the difficulties they faced, to take the difficult decision, at very short notice, to go ahead with the conference and allow a range of amazing research to be shared. Whether or not you were able to attend this conference, if you are interested in integrity in education, I can recommend this book to you. Congratulations to the editors and authors and all involved in putting together this very rich set of chapters on different aspects of academic integrity.

## Data Availability

N/A.

## Abbreviations

APEL: Accreditation for Prior Experiential Learning
ENAI: European Network for Academic Integrity
EU: European Union
ETPB: Extended theory of planned behaviour
IPPHEAE: Impact of Policies for Plagiarism in Higher Education across Europe
MAP: Moral system, attitude and pressure
OOXML: Open Office eXtensible Markup Language
PAEB: Plagiarism across Europe and Beyond Conference
UAE: United Arab Emirates
UK: United Kingdom
USA: United States of America
XML: eXtensible Markup Language
